# The Role of Topoisomerase II in DNA Repair and Recombination in *Arabidopsis thaliana*

**DOI:** 10.3390/ijms222313115

**Published:** 2021-12-04

**Authors:** Marina Martinez-Garcia, Charles I. White, F. Chris. H. Franklin, Eugenio Sanchez-Moran

**Affiliations:** 1 School of Biosciences, University of Birmingham, Edgbaston, Birmingham B15 2TT, UK; f.c.h.franklin@bham.ac.uk; 2 Génétique, Reproduction et Développement, Faculté de Médecine, UMR CNRS 6293—INSERM U1103—Université Clermont Auvergne, 28 Place Henri Dunant, 63001 Clermont-Ferrand, France; charles.white@uca.fr

**Keywords:** topoisomerase II, meiosis, homologous recombination, DNA repair, Arabidopsis

## Abstract

DNA entanglements and supercoiling arise frequently during normal DNA metabolism. DNA topoisomerases are highly conserved enzymes that resolve the topological problems that these structures create. Topoisomerase II (TOPII) releases topological stress in DNA by removing DNA supercoils through breaking the two DNA strands, passing a DNA duplex through the break and religating the broken strands. TOPII performs key DNA metabolic roles essential for DNA replication, chromosome condensation, heterochromatin metabolism, telomere disentanglement, centromere decatenation, transmission of crossover (CO) interference, interlock resolution and chromosome segregation in several model organisms. In this study, we reveal the endogenous role of *Arabidopsis thaliana* TOPII in normal root growth and cell cycle, and mitotic DNA repair via homologous recombination. Additionally, we show that the protein is required for meiotic DSB repair progression, but not for CO formation. We propose that TOPII might promote mitotic HR DNA repair by relieving stress needed for HR strand invasion and D-loop formation.

## 1. Introduction

DNA topoisomerases are highly conserved enzymes that resolve topological problems that arise during a wide range of DNA metabolic processes including DNA replication, DNA transcription, DNA repair, chromosome condensation, heterochromatin metabolism, telomere disentanglement, centromere decatenation, chromosome remodelling and segregation and meiotic interlock disentanglement [1,2,3,4,5,6,7,8]. Resolution of these topological challenges is achieved via a common mechanism that involves single-strand (type I class, TOPI and TOP3) or double-strand (type II class, TOPII) cleavage of the DNA helix, and a strand-passing step that releases the tension [9]. In both prokaryotes and eukaryotes, type II topoisomerases consist of a homodimer that requires ATP and Mg^+2^ to perform its catalytic activity: they bind to a region of the DNA called the G-segment and trap a second region of DNA, the T segment; ATP hydrolysis helps G-segment cleavage (DNA double-strand break formation), letting the T segment pass in between the broken ends. The DNA-protein crosslink created by the activity of TOPII, normally repaired by Tyrosyl-DNA-phosphodiesterase 2 [10], can cause DSBs. After releasing the DNA, the homodimer then returns to an open position. How this cycle, which affects DNA topology at a local level, can be coordinated with processes with a much greater scale of complexity, such as chromosome condensation, is still unknown [11].

During DNA replication, chromosomes are subject to mechanical stress that arises from DNA strand separation as they are copied. Helicases introduce negative supercoiling in the DNA when opening replicating forks, leading to the accumulation of positive DNA supercoiling ahead of the replicating fork bubble [12]. Whereas either TOPI or TOPII can liberate tension ahead of the fork, only TOPII can resolve the DNA precatenanes formed by fork swivelling between the new sister chromatids behind the replication bubble. TOPII also resolves any remaining DNA catenanes between sister chromatids during chromosome segregation [13,14].

Homologous recombination (HR) is an error-free DNA double-strand break (DSB) repair pathway that operates in response to DNA damage arising from damaged replication forks, exogenous DNA damage, and also, in the case of meiosis, in the repair of programmed SPO11-catalysed DSBs [15,16]. In mitotic cells HR preferentially uses the sister chromatid as the repair template. In the case of meiotic DSBs, both the sister chromatid and homologous chromosome are used as the repair template, although the repair pathway is strongly biased towards the latter [17,18]. DSB repair requires DNA strand invasion of the repair template, a process coordinated by the strand exchange protein RAD51 or in the case of meiosis, RAD51 and its meiosis-specific counterpart DMC1 [19,20]. Strand invasion results in strand-displacement followed by the formation of a D-loop, and subsequently, a Holliday Junction (HJ) intermediate, a cross-shaped structure involving four DNA strands switching partners [21]. Biochemical evidence indicates HJ formation requires negative supercoiling in the centre of this four-way DNA junction, and to compensate this tension, positive supercoiling is generated at both adjacent sides of the molecule [22]. Furthermore, stable strand invasion has also been reported to required negative supercoiling in the template strand and topoisomerases have been proposed to help achieve this conformation [23]. Evidence of a role for TOPII in HR mediated DSB repair was reported by Morotomi-Yano and colleagues [24]. They localised human TOPII to exogenous DSB sites and found increased sensitivity in *topIIβ* mutant HeLa cells to bleomycin together with reduced HR-dependent DSB repair. During meiosis in budding yeast, HR occurs near active transcription sites where Top2 also localises. Moreover, analysis of a *top2* mutant has found an excess of DSBs in late pachytene, consistent with a role for Top2 in meiotic HR [8].

Meiotic HR results in the repair of SPO11-programmed DSBs as either CO or non-CO products [25]. Studies have reported a role for TOPII in influencing the patterning of COs along chromosomes [26,27]. It is postulated that this occurs through TOPII influencing the mechanical properties of the chromosomes through an effect on the chromosome axis [27,28]. Indeed, several studies show that TOPII associates with chromosomes during prophase I in a variety of organisms including yeast and plants [7,29]. In the case of budding yeast, a meiosis specific knockout of Top2activity is associated with a change in the pattern of Zip3 protein foci, a marker for CO sites, at pachytene, suggesting a role for Top2 in mediating CO interference, the phenomenon that prevents COs occurring in close proximity [27]. However, the broader picture remains unclear, and whereas the study in yeast indicated a reduction in CO interference, treatment of mouse male meiocytes with the TOPII poison, etoposide, suggested an increase in CO interference [26].

Although it is assumed that many of the reported roles for TOPII will be conserved in plants, there are relatively few direct studies. The biochemical activity of TOPII has been reported in cauliflower buds and extracts of maize embryos [30,31]. *Nicotiana tabacum TOPII* complements a yeast *topII* mutant, indicating conservation. Additionally, the pattern of *NtTOPII* expression was suggestive of roles in chromosome segregation, condensation, DNA replication and transcription [32,33]. *Arabidopsis thaliana* TOPII has a very similar sequence to those of yeast and animals and is highly expressed in proliferating tissues [34,35]. In the case of meiosis, we have described a role for TOPII in meiotic chromosome condensation and interlock resolution [7].

In the present study, we investigate the role of TOPII in somatic and meiotic HR DNA repair in the model organism *Arabidopsis thaliana*. We describe abnormal root development in *topII-1* mutants, exhibiting high levels of cell death, probably stemming from the accumulation of unrepaired DSBs arising from DNA replication problems during mitosis. However, the analysis of pollen mother cells indicates that replication damage does not accumulate or persist to the same extent in meiosis. Arabidopsis *topII* mutants are sensitive to γ irradiation and mitomycin C, confirming the implication of TOPII in the maintenance of the genome integrity through development. Moreover, we observe a delay in DSB repair during meiosis, though generally, normal meiotic recombination levels and CO patterning. In light of these results, we propose a model in which TOPII would be needed to release tension created by HR DNA repair, by solving the positive supercoiling accumulated around those regions.

## 2. Results

### 2.1. TOPII Plays an Important Role in Mitotic DNA Replication

**Figure 1:**
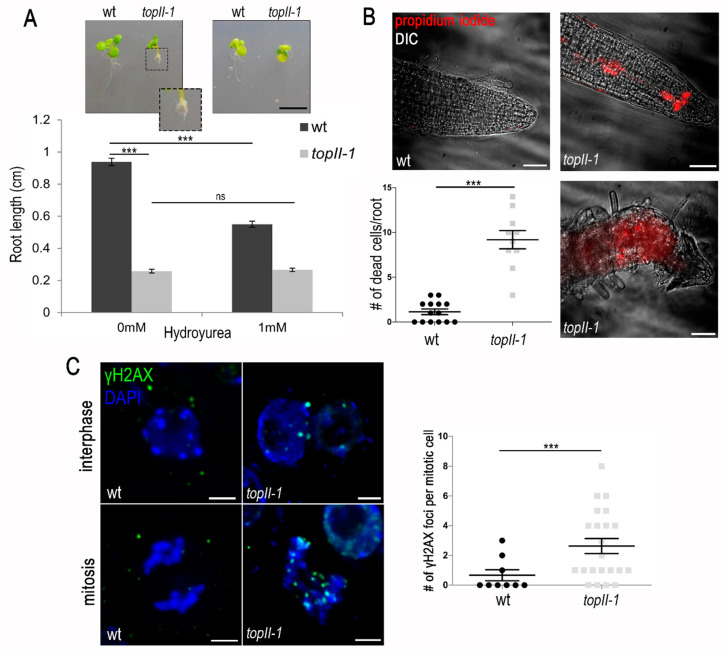
TOPII is needed for early root mitotic growth. (**A**) wt and *topII-1* root growth and sensitivity to HU (*t*-test). (**B**) Root cell death assessment by propidium iodide staining (red) (Fisher’s exact test). Scale bar: 50 µm. (**C**) γH2AX staining (green) in mitotic interphase and cell divisions in wt and *topII-1* (Mann–Whitney U-test). Scale bar: 5 µm. Bars: mean ± Scheme 0. *p*-values: *** <0.001; ns: not significant.

One possible origin of the previously observed mitotic chromosome bridges in *topII-1* [7] could be problems during DNA replication. This has been reported in other organisms where TOPII has been mutated [14] or where its catalytic activity was blocked with inhibitors [33]. To investigate this, we assessed early root development in seedlings, since many cells in this tissue are undergoing DNA replication and mitosis. The *topII-1* line displayed extremely short roots 14 days after sowing (0.26 ± 0.01 cm, *n* = 50; wild-type 0.94 ± 0.02 cm *n* = 50, *t*-test *p* < 0.001, Figure 1A). To test if TOPII has a role in the fork-stalled checkpoint pathway, wild-type and *topII-1* seeds were grown in vertical MS plates with 1 mM of hydroxyurea (HU) (Figure 1A). HU delays DNA replication by limiting the amount of dNTPs available, and therefore, stalling replication forks [36]. As expected, wild-type root length was affected by the treatment, showing a reduction from 0.94 ± 0.16 cm to 0.55 ± 0.02 cm (*n* = 50, *t*-test *p* < 0.001). However, HU treatment did not result in an additional reduction in root growth in the *topII-1* mutant (0.26 ± 0.01 cm *n* = 50 vs. 0.27 ± 0.01 cm *n* = 46 after HU, *t*-test *p* = 0.613). Thus, root growth in the mutant is not further compromised by the inhibitor treatment.

To determine the level of tissue damage arising from the malfunctioning of TOPII, *topII-1* roots were stained with propidium iodide (PI). PI is a fluorochrome that binds to DNA but is unable to penetrate the cytoplasmic membrane of living cells, and thus, only stains nuclei of dead cells. Wild-type plants showed normal development of a long primary root with little evidence of cell death (1.14 dead cells per meristem, *n* = 15, Figure 1B). In contrast, *topII-1* plants developed multiple short roots. These roots initially showed high levels of cell death (9.20 dead cells per meristem, Fisher’s exact test *p* < 0.001, *n* = 10). Most roots showed abnormal cells and very long root hairs that developed into deformed roots (*n* = 12 of 22) (Figure 1B).

To investigate whether collapsed replication forks were creating transient DSB in root meristem cells, we used immunolocalisation of γH2AX, which marks the sites of DSBs [37]. Root interphase nuclei showed a dramatic 6.5-fold increase in the number of cells with γH2AX foci in *topII-1* (20.85% *n* = 528, vs. wild-type 3.20% *n* = 219, Pearson Chi-square *p* < 0.001, Figure 1C). Surprisingly, some breaks remained unrepaired even though the cell cycle progressed, as γH2AX foci persisted to metaphase and anaphase stages (*topII-1* 2.64 ± 0.50 foci per cell, *n* = 22; wild-type 0.67 ± 0.37, *n* = 9; Mann–Whitney U test *p* = 0.014, Figure 1C). Chromatin bridges during anaphase were found in 14.4% of *topII-1* tapetum cells (*n* = 194) in flower buds, compared to zero in wild-type (*n* = 143, *p* < 0.001, Figure S1D).

In contrast to mitotic cells, during meiosis the number of replication-associated DSBs (that is, as distinct from programmed SPO11-catalysed DSBs in meiotic prophase I) in *topII-1* was indistinguishable from wild-type [7]. The number of γH2AX foci in a *topII-1 spo11-2-2* double mutant was not significantly different to those in a *spo11-2-2* single mutant (13.79 ± 1.73 *n* = 19, 9.14 ± 0.63 *n* = 14, respectively, Mann–Whitney U test *p* = 0.181, Figure S1A). This suggests that pre-meiotic DNA replication may have a stronger DNA damage checkpoint and most problematic cells are prevented entry into the meiotic program. Nevertheless, cells with minor replication problems do on occasion seem to enter meiosis, as in a few instances, we observed cells at anaphase II with bridges between sister chromatids in the *topII-1 x spo11-2-2* double mutant (Figure S1B).

### 2.2. HR-Mediated DNA Repair Problems Are Observed in TopII-1

**Figure 2:**
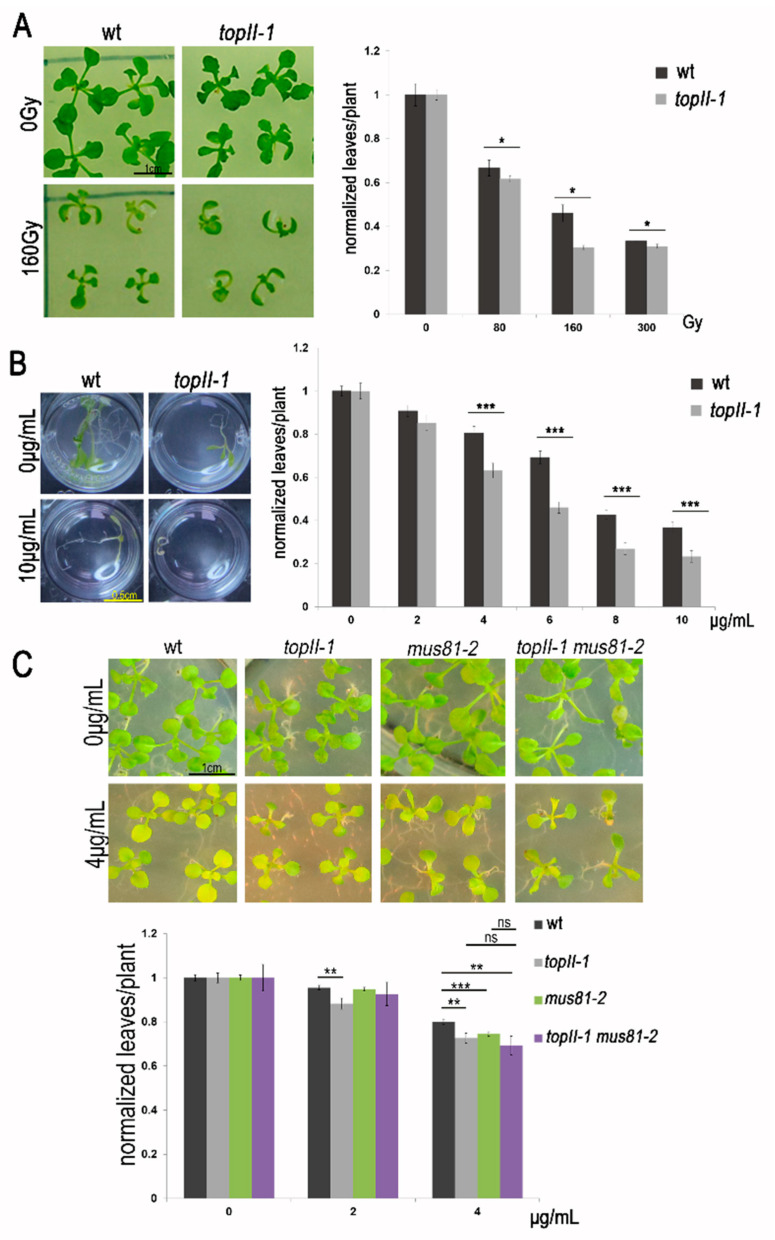
*topII-1* is sensitive to DNA damage dependent on mitotic HR. (**A**) Effects of γ irradiation (0, 80, 160 and 300 Gy) on wt and *topII-1* somatic seedling development. (**B**) Mitomycin C liquid exposure effects on somatic development on wt and *topII-1* plants. (**C**) Mitomycin C exposure effects on somatic development of wt, *topII-1, mus81-2* and *topII-1 x mus81-2* seedlings. Bars: mean ± standard error of the mean. *t*-student test *p*-values: * <0.05, ** <0.01, *** <0.001; ns: not significant.

Detection of elevated levels of DSBs in *topII-1* root cells led us to investigate a possible role for TOPII in the mitotic HR DSB repair pathway. Hence, we exposed the mutant plants to a variety of genotoxic agents. Ionising radiation (IR) creates several forms of DNA damage directly or via reactive oxygen species. IR produces a mixture of single and complex double-stranded DNA breaks, the latter repaired by either NHEJ or HR [38,39]. Seeds of *topII-1* treated with different doses of IR showed significantly different growth compared to wild-type from 80 Gy to 300 Gy (*n* = 20 for Ws and *n* = 80 for *topII-1 per* dose, *t*-student *p* < 0.05, Figure 2A). The drug mitomycin C (MMC) forms DNA inter-strand crosslinks that can impede the progression of DNA replication. Inter-strand crosslinks are generally repaired by the nucleotide excision repair pathway, but during S-phase and G2 they are preferentially repaired by HR via the formation of DSBs [40,41]. Treated *topII-1* plants produced significantly fewer leaves per plant than wild-type with MMC doses of 4 to 10 µg/mL (*n* = 100 per line per dose, *t*-student *p* < 0.001, Figure 2B). Overall, these results suggest that *topII-1* mutation results in problems in the repair of DSBs via the somatic HR pathway. To further investigate this, we produced a *topII-1 mus81-2* double mutant. MUS81 accounts for less than 15% of COs during meiosis Arabidopsis [42], but plays a primary role during somatic HR [43]. To evaluate whether HR defects in *topII-1* are due to a defect in the MUS81 repair pathway, *topII-1*, *mus81-2* and *topII-1 x mus81-2* double mutant plants were sown in the same plates and exposed to two doses of MMC, as previously described [43]. While all three lines were significantly different compared to wild-type (*n* = 140–170 per line per dose, *t*-student *p* < 0.05), plants from the double mutant showed equivalent sensitivity to MMC in comparison to the individual mutants (*p*-value > 0.2 in all *t*-tests, Figure 2C). Moreover, similar results were obtained when exposing single *topII-1* and *mus81-2* and double mutant plants to the genotoxic agent cisplatin. Cisplatin produces DNA intra-strand crosslinks that are mostly repaired by nucleotide excision repair pathway and occasionally through HR [44,45]. The number of leaves in both the single and the double mutant plants was significantly different from the wild-type (*p* < 0.05), but the double *topII-1 mus81-2* was only significantly different from the *topII-1* line (*p* = 0.006, Figure S1C). The formation of anaphase chromatin bridges in the double mutant *topII-1 mus81-2* was equivalent to *topII-1* levels (0.173 bridges/cell and *n* = 260, 0.144 bridges/cell and *n* = 194, respectively, *p* = 0.44, Figure S1D) and comparable to previously published levels in *mus81-2* mutant plants (0.3, *n* = 295, [46]). This suggests TOPII might be acting in the same pathway as MUS81, since no additive effects were found in the double mutant in the number of anaphase bridges, when exposed to MMC, and was not significantly different when exposed to cisplatin.

### 2.3. The Dynamics of Meiotic DSB Repair Are Affected in TopII-1

**Figure 3:**
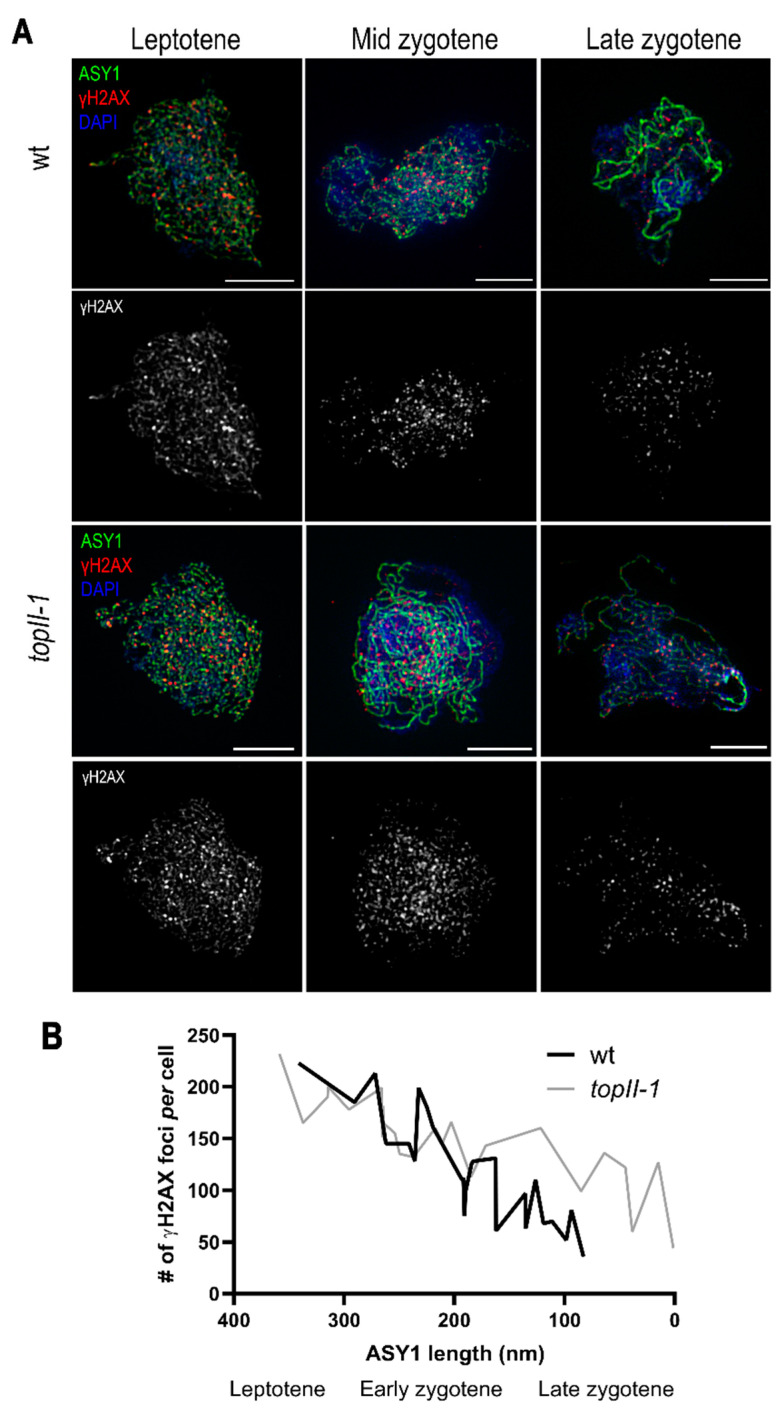
TOPII is needed for the timely progression of meiotic DSB repair. (**A**) ASY1 (green) and γH2AX (red) staining of early prophase I wt and *topII-1* meiocytes. (**B**) γH2AX foci distribution per cell vs. ASY1 length (nm). Scale bars: 5 µm.

We have previously shown that despite an increased incidence of chromosome interlocks during meiosis in *topII-1*, CO formation via the HR pathway is completed and the plants are fertile [7]. However, as HR in mitotic cells is affected in *topII-1*, we investigated if progression of DSB repair during meiotic prophase I was normal. Meiotic progression in relation to DSB repair was monitored in chromosome spread preparations of pollen mother cells (PMCs) using dual-immunolocalisation of the chromosome axis component ASY1 and γH2AX foci to mark DSBs [47] (Figure 3A).

At early leptotene, ASY1 forms a linear signal along each chromosome. At the onset of zygotene, the synaptonemal complex (SC) begins to polymerise between the aligned homologous chromosomes and this can be monitored by immunolocalisation of the ZYP1 protein. As ZYP1 polymerises along the homologous chromosomes, the ASY1 signal becomes progressively diffuse and with completion of the SC at pachytene, linear ASY1 stretches are no longer observed [47,48]. The relation between ASY1 fibre length and numbers of γH2AX foci approximates an exponential decay equation: (y = [(1/2)]^x = e^(−Ln(x)) = y_o e^(−λt); λ = decay constant, t = time) [49] (Figure 3B). Transforming the variables with a natural logarithm to produce a linear regression (y = b + mx) (Lny = Lny_o-λt), the equation fits a linear distribution in both cases, although without a high R^2^ value (Ws R^2^ = 0.73, *topII-1* R^2^ = 0.70). The slopes denote the speed of disappearance of γH2AX foci (“repair of DSBs”) relative to the reduction in length of the ASY1 axis signal. Slopes were compared by ‘regression slope *t*-test’ and were found to be significantly different (Ws 1.103 and *topII-1* 0.233, t = 6.17, *p* < 0.001). Therefore, the lower slope in *topII-1* points to a delay in DSB repair in the mutant. Thus, the defect in TOPII activity in the mutant reduces the rate of DSB repair via HR in meiocytes.

### 2.4. Participation of TOPII in Meiotic CO Formation

TOPII has been proposed as a regulator of DNA tension during the mitotic cycle and meiotic prophase I [28]. The role of TOPII in interlock resolution in meiotic chromosomes is proposed to be a reflection of this activity [7]. This is also thought to be the case for its participation in modulating crossover (CO) interference in budding yeast, the mechanism which results in the patterned spacing of COs along chromosomes [27]. Therefore, we investigated if the *topII-1* mutation would also influence CO interference in plants.

**Figure 4:**
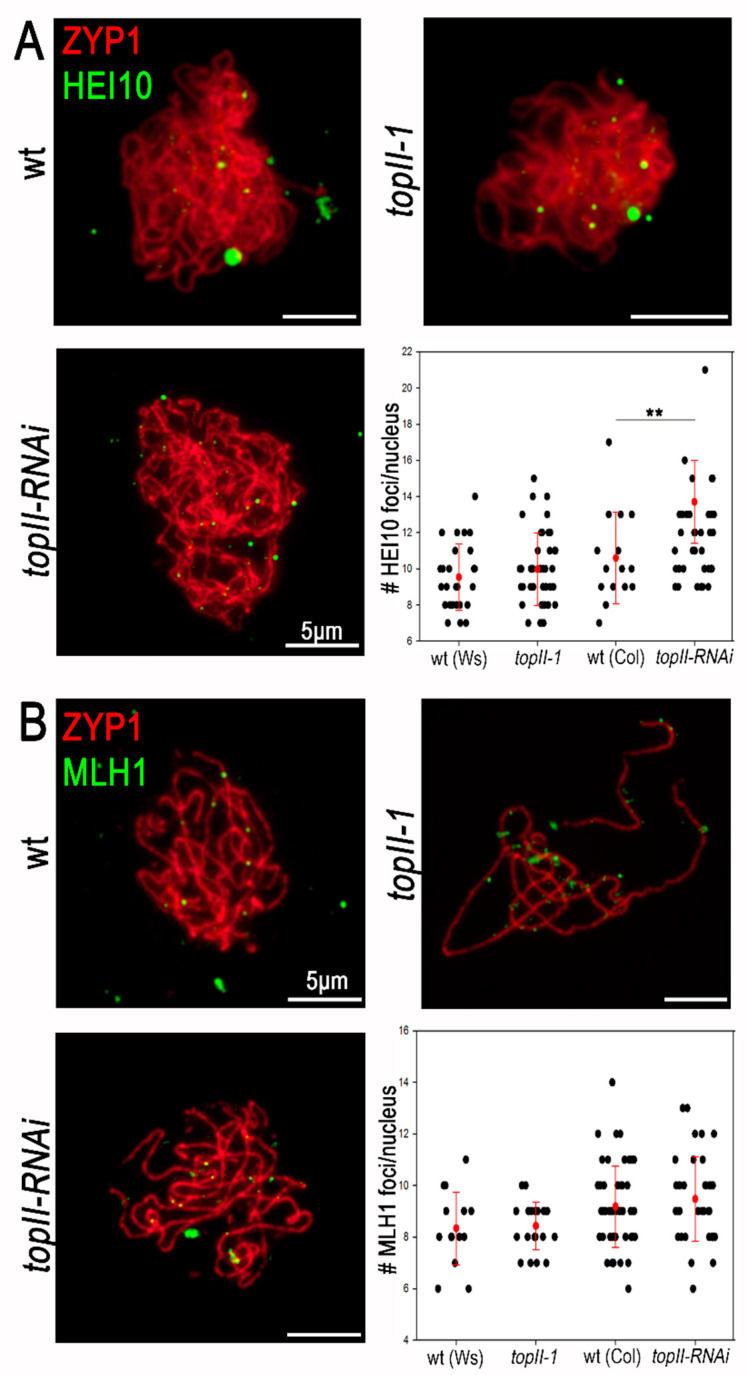
Meiotic CO designation and maturation appears normal in *topII* plants. (**A**) HEI10 (green) and ASY1 (red) staining of wt, *topII-1* and *topII-RNAi* zygotene cells and HEI10 foci distribution per cell. (**B**) MLH1 (green) and ZYP1 (red) staining of wt, *topII-1* and *topII-RNAi* pachytene cells and MLH1 foci distribution per cell. Bars: mean ± standard deviation. Mann–Whitney U-test, *p*-value: ** <0.01.

The HEI10 protein (a homologue of yeast Zip3) binds recombination intermediates during early prophase I [50,51]. As prophase I progresses, the number of HEI10 foci gradually reduces until only those marking CO designated intermediates remain. This process is proposed to reflect the propagation of an interference signal that prevents closely spaced CO designation events [51]. Immunolocalisation of HEI10 in chromosome spreads from PMCs at pachytene revealed no significant difference in the number of HEI10 foci in *topII-1* compared to wild-type (9.54 ± 0.56 *n* = 26, *topII-1* 9.98 ± 0.31 *n* = 41, Mann–Whitney U test *p* = 0.393, Figure 4A). CO maturation, assessed by MLH1 immunostaining at pachytene, also appeared normal in *topII-1* plants (8.33 ± 0.36 *n* = 15, *topII-1* 8.42 ± 0.20 *n* = 21, *p* = 0.776 Figure 4B).

Although the hypomorphic *topII-1* mutation did not appear to influence CO patterning, this could be due to residual activity of the mutant protein. Therefore, we repeated the analysis using a meiosis-specific *topII-RNAi* line, which has previously been shown to accumulate increased levels of meiotic interlocks compared to *topII-1*, due to reduced levels of TOPII protein [7]. Immunolocalisation of HEI10 foci showed a moderate increase in the number of foci at pachytene compared to wild-type (Col) (10.60 ± 0.65 *n* = 15, *topII-RNAi* 13.25 ± 0.34 *n* = 16, *p* = 0.002, Figure 4A). This could suggest a possible increase in CO designation events; however, this difference is small and numbers of MLH1 foci did not show any detectable increase (MLH1: 9.18 ± 0.22 *n* = 50, *topII-RNAi* 9.47 ± 0.27 *n* = 36, *p* = 0.373 Figure 4B). Thus, it seems more likely that the accumulation of chromosome interlocks and entanglements in this line could be influencing normal prophase I progression.

**Figure 5:**
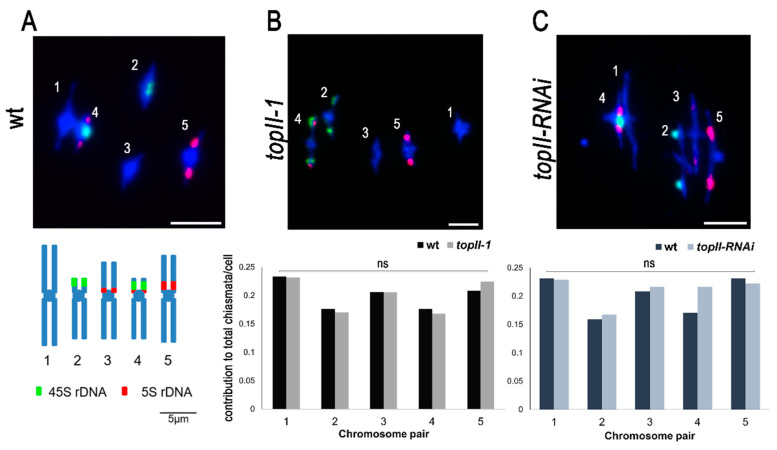
Chiasma frequency and distribution are not modified in *topII* plants. (**A**) Wild-type metaphase I nucleus and mitotic ideogram with the localisation of rDNA FISH probes (45S in green and 5S in red). Scale bars: 5 µm. (**B**) *topII-1* representative metaphase I nucleus and graph showing chiasma distributions per chromosome in wt (Ws) and *topII-1*. (**C**) *topII-RNAi* representative metaphase I meioses and graph showing chiasma distribution per chromosome in wt (Col) and *topII-RNAi*. Mann–Whitney U-test, ns: not significant.

Finally, CO numbers were estimated by determining chiasma frequencies at metaphase I. Again, no difference between wt and *topII-1* plants was found for either the chiasma frequency (8.35 ± 0.14 *n* = 57, 8.13 ± 0.17 *n* = 52, respectively, Mann–Whitney U test *p* = 0.376, Figure 5A,B) or the chiasma distribution between different homologous chromosome pairs (Mann–Whitney U test *p* > 0.1, Figure 5A,B). Similar results were obtained using the *topII-RNAi* with no significant differences found in mean numbers of chiasmata per meiosis (wild type 8.65 ± 0.16 *n* = 40, *topII-RNAi* 9.04 ± 0.15 *n* = 47, Mann–Whitney U test *p* = 0.079, Figure 5C) or the chiasma distribution between different pairs of homologous chromosomes analysed by FISH (Mann–Whitney U test *p* > 0.1, Figure 5C).

## 3. Discussion

### 3.1. Mitotic DNA Replication Is Affected More Than Meiotic DNA Replication in TopII-1

The role of TOPII during DNA replication has been extensively studied in other organisms [14]. The growth defect observed in *topII-1* seedling roots combined with evidence of a 6.5-fold increase in DSBs in root meristem cells is consistent with an important role for TOPII during mitotic replication in *A. thaliana*. In a previous study, wild-type Arabidopsis seedlings grown in the presence of the replication inhibitors hydroxyurea (HU) and aphidicolin showed a reduction in root growth of around 40% [52]. However, the *topII-1* seedling root phenotype is more extreme, and notably, treatment with HU does not lead to any obvious additional effect on root growth in *topII-1* seedlings. The *topII-1* phenotype is more similar to the response of mutant plants of the replication checkpoint kinase ATR following exposure to the replication inhibitors, which results in mitotic catastrophe leading to shortened roots, extended root hairs, abnormal cell morphology and cell death [52]. We propose that in the case of *topII-1,* where TOPII is not completely functional, accumulation of positive supercoiling in the vicinity of replication forks could lead to their collapse and consequent delay in replication and that, in this context, lowering the concentration of dNTPs by application of HU, imposes no additional delay in growth.

Despite the root defect observed at the seedling stage, *topII-1* plants do grow and develop to reach maturity [7]. This suggests that the DNA damage in *topII-1* is sufficiently well dealt with by alternative pathways, such as endoreduplication or programmed cell death, to allow growth to proceed [53,54]. That said, detection of γH2AX foci in root meristem cells at metaphase and anaphase stages shows that at least some DSBs remain unrepaired throughout mitosis.

In contrast to mitosis, the impact of *topII-1* on pre-meiotic DNA replication appears less severe, with notably no significant difference in the number of DSBs in a *topII-1 spo11-2-2* double mutant compared to *spo11-2-2*. The TOPII defect in *topII-1*, thus, does not lead to the presence of additional unrepaired DSBs in meiotic prophase I. This has also been reported for Top2 in budding yeast [8]. In addition, a previous study showed that while the TOPII inhibitor etoposide inhibited replication of mitotic S-phase cells, it was less effective when added to premeiotic cells [55]. Analysis of an Arabidopsis *FASCIATA* mutant also suggested that in comparison to mitotic S-phase, meiotic DNA replication may be more tolerant to perturbation [56,57]. Although the basis for this apparent difference is unclear, meiotic replication is reported to take significantly longer in a variety of species, such as budding yeast, lily and Arabidopsis [58,59,60,61]. It is proposed that this additional time may be to accommodate the loading of meiosis-specific proteins required for meiotic chromosome organisation and homologous recombination [59]. In the case of *topII-1* the additional time spent in meiotic S-phase may also allow the resolution of replication problems.

### 3.2. CO Formation Appears Normal in TopII-1 and TopII-RNAi Plants

In budding yeast meiosis, specific depletion of Top2 is reported to lead to a reduction in CO interference that manifests in a change in the distribution of foci of the HEI10 ortholog Zip3 along homologous chromosome pairs [27]. Our analysis of HEI10 foci in *topII-1* did not detect any significant difference from wild-type plants. Similarly, the number of MLH1 foci at pachytene and chiasmata at metaphase I appeared unchanged. In the case of the *topII-RNAi* line a slight increase in HEI10 foci was detected at pachytene, but no accompanying change in MLH1 foci nor of chiasmata was observed. Thus, although the increase in HEI10 foci could indicate an effect on interference in *topII-RNAi*, it appears more likely that, in this line, a delay in DSB repair dynamics similar to that found in *topII-1* might underly this observation. Previous work has shown that at late leptotene there are around 165 HEI10 foci and that this gradually decreases as prophase I progresses until around 10 remain at late prophase I [50,62]. Hence, even a slight delay in the repair dynamics could influence the number of foci detected at intermediate stages of prophase I. These observations could suggest that in contrast to budding yeast, TOPII does not play a role in CO interference in Arabidopsis. However, in all probability, a residual level of TOPII activity remains in both *topII-1* and *topII-RNAi* lines, since repeated attempts to isolate a *TOPII* null mutant indicate that total loss of activity is lethal [7,63]. Thus, the possibility remains that residual TOPII activity in these lines is sufficient to ensure normal CO patterning or that any change is too subtle to be detected by the methods employed in this study.

### 3.3. Mitotic and Meiotic DSBs Can Remain Unrepaired in TopII-1

**Figure 6:**
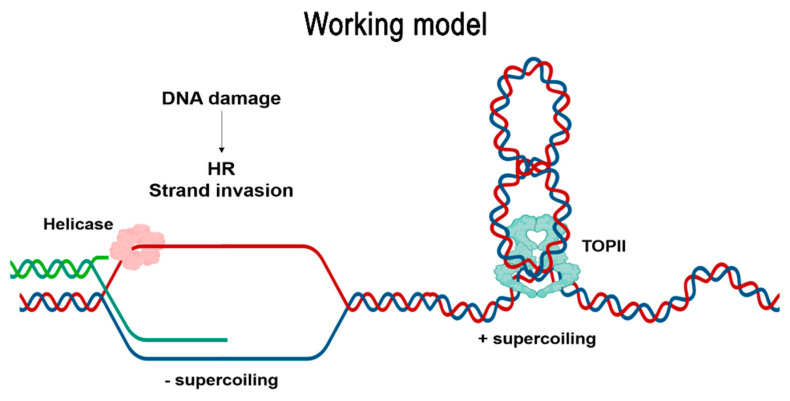
Working model of TOPII participation in the resolution of topological stress generated during HR. When DNA damage is repaired by homologous recombination, the DNA double-helix of the repair template needs to be uncoiled by the action of helicases to form a D-loop, thereby enabling strand invasion. This generates negative supercoiling of the DNA, which in turn is compensated by positive supercoiling at both ends of the D-loop. TOPII potentially relieves part of the mechanical stress generated in the vicinity of the HR intermediate, facilitating the repair process.

That *topII-1* exhibited persistent DSBs in both mitosis and meiosis suggests an additional role for TOPII in DNA repair. The hypersensitivity of *topII-1* to MMC and, to a lesser extent, γ irradiation, supports this hypothesis. A slower rate of DSB repair of *topII-1* would also accord with the presence of chromosome bridges and constrictions observed at anaphase. Persistence of DSBs during late pachytene in *top2* mutants has been described in yeast [8]. In mammals, TOPIIβ has been shown to localise to exogenous DSBs and HeLa cells lacking this TOPII isoform are sensitive to bleomycin (a radiomimetic cancer drug that produces DSBs associated with HR repair) [24,64]. In line with our results (Figure S1C), Arabidopsis plants treated with the TOPII poison etoposide showed hypersensitivity to cisplatin in a study describing the phenotype of TOPII binding protein 1-mutants [65]. Since repair of MMC DNA adducts and complex DSBs arising from γ irradiation involves HR [40,41,66], TOPII could be playing a role in this pathway. Although it is difficult to see a direct involvement of the protein, it seems reasonable to suppose that TOPII could be releasing torsional stress created by HR DNA repair (Figure 6).

TOPII is able to recognise, bind and cut in vitro complex DNA structures such as four-way junctions (i.e., HJs) [67,68]. HJ formation requires a certain level of negative supercoiling in order to exchange DNA strands, and subsequently, forms positive supercoiling at either adjacent end to compensate [22]. When the DNA double helix is negatively supercoiled, the ssDNA filament loaded with RecA (the bacterial equivalent to RAD51/DMC1) is more efficient in invading and forming a joint molecule [69,70]. Lu and Li [23] proposed the participation of topoisomerases in releasing this stress, because DNA substrates with a nick allow RecA strand exchange four times more efficiently than in the absence of a nick. In fact, topoisomerase I has been shown to physically bind RecA and help the homology search [71]. Moreover, TOPBP1 (Topoisomerase II binding protein 1) controls the binding of either 53BP1 (which inhibits HR) or BRCA1 (which promotes HR) to the ssDNA [6]. This points to the possibility that TOPII activity is complementary to TOPI in solving the torsional stress needed for the HR pathway.

Taking together our observations that programmed meiotic DSBs in *topII-1* persist into late zygotene, meiotic anaphase I bridges are SPO11-dependent and the MMC sensitivity in a *topII* *mus81* double mutant is similar to that in the single mutants suggests a complex model in which pleiotropic activity of TOPII could be influencing several processes in HR (Figure 6). If TOPII is involved in the release of the topological stress in DNA strand-invasion intermediate structures (Figure 6), how this articulates with chromatin structure in a context with nucleosomes in eukaryotes will be of particular interest.

## 4. Materials and Methods

### 4.1. Plant Material

*Arabidopsis thaliana* plants were supplied by the NASC (Nottingham Arabidopsis Stock Centre) and IJBP Arabidopsis Stock Centre. Arabidopsis ecotype Col-0 and Ws-0 were used as a control depending on the genetic background of the mutant lines. Plants were grown in a greenhouse with controlled temperature and humidity (20 °C and 60%, respectively) and summer photoperiod conditions (16 h light and 8 h darkness), and sown in a mixture of 4 parts M3 compost [72]. Mutant lines used in this study were the hypomorphic T-DNA insertion mutant *topII-1* and the meiosis-specific *topII-RNAi* line controlled by a DMC1 promoter [7], and the T-DNA mutant lines *spo11-2-2* [73], *mus81-2* [42] and *mlh3-1* [74].

### 4.2. Plant Growth and Genotoxicity Experiments

Plants were grown in MS medium [75], 1% sucrose and phytoagar (pH = 5.7) after sterilisation [76]. Sensitivity to dNTP shortage by hydroxyurea (HU) treatment was performed sowing sterile seeds in vertical square Petri dishes in 0 and 1 mM HU [52], and measuring root length 3 weeks after sowing with Fiji (ImageJ) measuring tool [77]. Cell death analysis was performed staining Arabidopsis roots, after 5 days of germination in sterile conditions, with Propidium iodide (PI) 5 µg/mL in water as loss of cell integrity allows its selective uptake [78]. Root tips were cut and mounted in a drop of water in a slide and images were taken using a Zeiss Axiolmager Z1 (Carl Zeiss AG) epifluorescence microscope. Z-stack projections of the roots were used to assess the number of dead areas per root [79].

Sensitivity to ionising radiation was performed using an X-ray machine with a cobalt source [46]. Plants were exposed after germination to 0, 80, 160 and 300 Gy. The number of leaves was assessed after 14 days growth in long day conditions at 20 °C. Mitomycin C sensitivity was assessed in liquid culture [7] at 0, 2, 4, 6, 8 and 10 µg/mL for wild-type and *topII-1*, and in MS plates at 0, 2 and 4 µg/mL for wild-type, *topII-1, mus81-2* and *topII-1 mus81-2*. Resistance to cisplatin treatment was evaluated in MS plates at 0, 10 and 15 µM for wild-type, *topII-1, mus81-2* and *topII-1 mus81-2* [43].

### 4.3. Cytogenetic Techniques

Five-day-old Arabidopsis plantlets had roots dissected and squashed onto slides. Immunostaining with anti-γH2AX [39] and Alexa 568 and mounted in Vectashield (Vector laboratories, Burlingame, US) and DAPI. Z-stacks were acquired using an AxioImager Z1 microscope Zeiss #49 and #47HE filter sets, processed and deconvolved using Zeiss Axiovision software as previously described [39,80].

Fresh inflorescences from *A. thaliana* plants were collected in humid chambers at 4 °C following the protocol of Armstrong and colleagues (2009) with modifications for immunostaining of proteins in pollen mother cells [81]. The meiotic stage of each bud was analysed under a phase-contrast microscope by staining with lacto-propionic orcein (LPO). A total of 5–10 buds of the desired size were put into 10 µL of enzyme mix (0.4% cytohelicase, 1% polyvinylpyrrolidone) on a poly-lysine slide (Thermo Fisher) and lysed using 1% Lipsol (SciLabware, Stroke on Trent, UK). Ice-cold 4% paraformaldehyde was added to the slide and left to dry for 1–2 h. Antibody incubation was performed in a humid chamber at 4 °C: α-ASY1 rat 1:500 [48], α-γH2AX rabbit 1:600 [39], α-HEI10 rabbit [62], α-ZYP1 rat 1:500 [82] and α-MLH1 rabbit [83]. Secondary antibody incubation was performed for 1 h at 37 °C (α-rat TexasRed, 1:200; α-rat FITC, 1:50; α-rabbitCy3, 1:100; α-rabbit FITC, 1:50) and co-stained with DAPI in Vectashield mounting media.

Chiasma scoring was performed on DAPI-stained chromosome spreads with Fluorescent In-situ Hybridisation (FISH) rDNA probes [84]. Floral buds conserved in 3:1 fixative (three parts absolute ethanol and one part glacial acetic acid) were dissected and digested for 2 h at 37 °C in a humid chamber. Slides were made from a single bud to have similar meiotic stages, due to anther synchrony. The cell suspension plus 10 µL of 60% acetic acid was placed on a hot plate at 45 °C for a minute and stirred with a dissecting needle. Dried slides were stained with DAPI and mounted in Vectashield [85]. Somatic anaphase bridges were analysed in tapetum cells using the same DAPI-stained spreads technique. Slides with metaphase I cells were subjected to DNA denaturation and hybridisation with a combination of DNA probes of 45S rDNA (from plasmid pTa71) and 5S rDNA (from plasmid pCT4.2) labelled with digoxigenin-dUTP or biotin-dUTP, and stained with anti-digoxigenin-FITC and avidin-Cy3 [76,84]. Images were captured using a 0.1 µm separation of each Z-stack using a Nikon i90 fluorescent microscope equipped with a Nikon DS-Qi1Mc digital camera and plan Apochromat VC 100x 1.40 N.A. oil ∞/0.17 differential interference contrast N2 objective. NIS Elements software (Nikon) was used to acquired images and deconvolve in the case of meiotic α-γH2AX staining (“MexicanHat”). Scoring HEI10 and MLH1 foci was performed using the “GaussLaplace Sharpen” (power 1.5). Blind scoring was performed by randomisation of images. Fiji (ImageJ) was used for adding a scale bar, transforming .nd2 images to 8-bit .tiff, inverting the colours and adjusting the brightness and contrast.

### 4.4. Statistical and Graphic Methods

Linear regression and basic bar charts were generated using Microsoft Excel 2016. Regression slope *t*-test was performed using the online tool (http://www.danielsoper.com/statcalc/calculator.aspx?id=103, accessed on 15 September 2017). Box plots were created with Sigmaplot 13. Statistical analysis was performed using IBM SPSS 22. A *t*-test was used to analyse differences of continuous data (i.e., root length). The non-parametric Mann–Whitney U-test was used to analyse differences of non-continuous data (i.e., chiasma frequency, HEI10 or MLH1 foci per cell). The Fisher-exact or Chi-square test was performed to compare proportions (i.e., dead cells per meristem). Data were represented as mean ± standard error of the mean, unless specified. The working model figure was created using BioRender.

## Supplementary Materials

The following are available online at https://www.mdpi.com/article/10.3390/ijms222313115/s1.
