# Supplementary material for: The Role of Topoisomerase II in DNA Repair and Recombination in Arabidopsis thaliana
Source: Int J Mol Sci. 2021 Dec 4;22(23):13115. doi: 10.3390/ijms222313115 (PMC8658145; doi:10.3390/ijms222313115)
Supplement: Supplementary file 1 [file ijms-22-13115-s001.zip › ijms-1494240-supplementary.pdf]

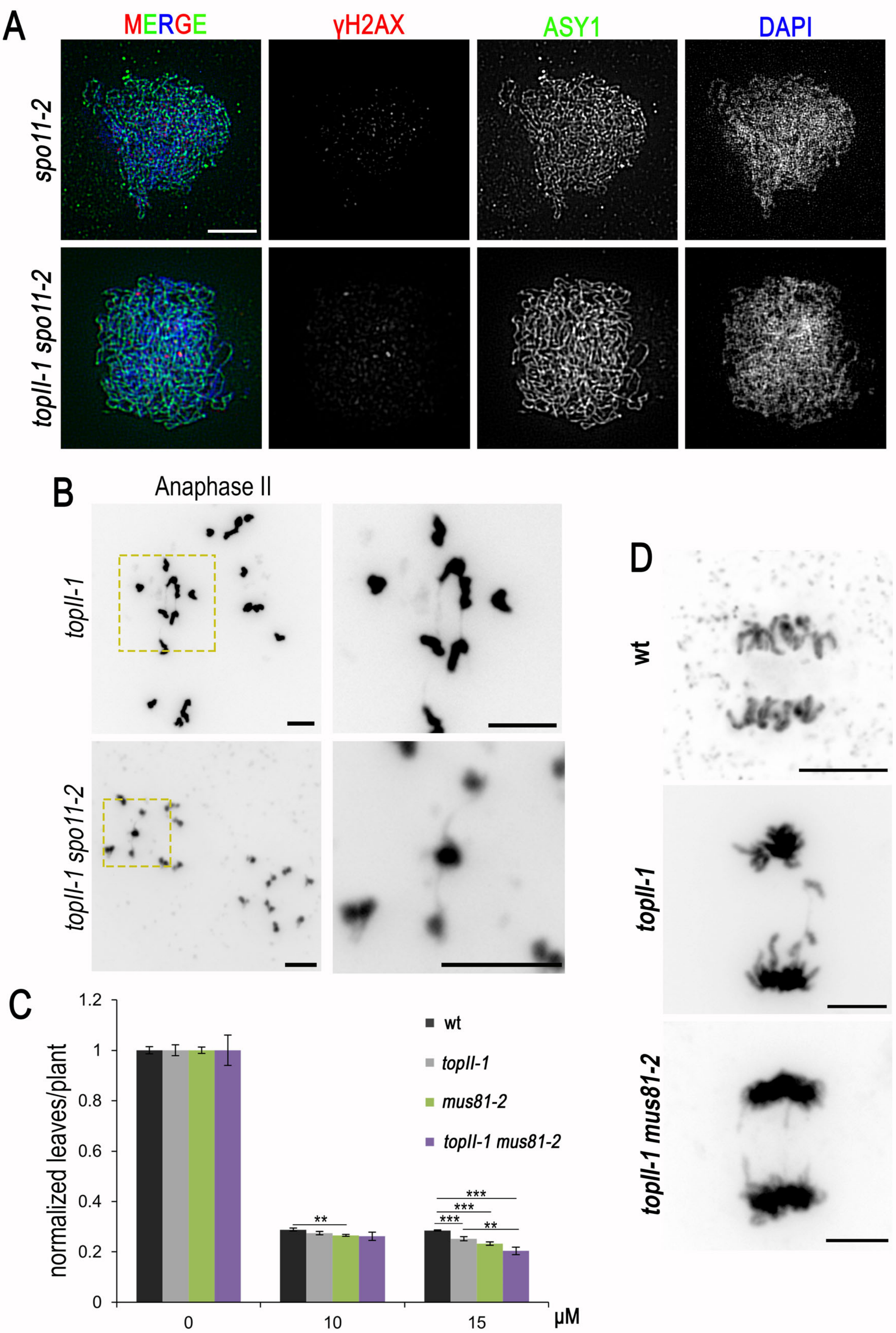

Supplementary Figure S1. A. γH2AX (red) and ASY1 (green) staining in leptotene cells of *spo11-2* and *topII-1 spo11-2*. **B.** Examples of anaphase II cells with chromatin bridges in *topII-1* and *topII-1 spo11-2*. **C.** Cisplatin exposure effects on somatic development of wt, *topII-1*, *mus81-2* and *topII-1 mus81-2* seedlings. T-test p-value: **<0.01, ***<0.001. **D.** Representative images of mitotic anaphase cells with chromatin bridges in wt, *topII-1* and *topII-1 mus81-2*. Scale bars: 5μm.
